# 2,4-Bis(2-eth­oxy­phen­yl)-7-methyl-3-aza­bicyclo­[3.3.1]nonan-9-one

**DOI:** 10.1107/S1600536811018472

**Published:** 2011-05-20

**Authors:** P. Parthiban, V. Ramkumar, Dong Ho Park, Yeon Tae Jeong

**Affiliations:** a Department of Image Science and Engineering, Pukyong National University, Busan 608 737, Republic of Korea; b Department of Chemistry, Indian Institute of Technology Madras, Chennai, Tamilnadu, India; c Department of Biomedicinal Chemistry, Inje University, Gimhae, Gyeongnam 621 749, Republic of Korea

## Abstract

The crystal structure of the title compound, C_25_H_31_NO_3_, exists in a twin-chair conformation with an equatorial orientation of the *ortho*-eth­oxy­phenyl groups. According to Cremer and Pople [Cremer & Pople (1975 ▶), *J. Am. Chem. Soc*. **97**, 1354–1358], both the piperidone and cyclo­hexa­none rings are significantly puckered with total puckering amplitutdes *Q*_T_ of 0.5889 (18) and 0.554 (2) Å, respectively. The *ortho*-eth­oxy­phenyl groups are located on either side of the secondary amino group and make a dihedral angle of 12.41 (4)° with respect to each other. The methyl group on the cyclo­hexa­none part occupies an exocyclic equatorial disposition. The crystal packing is stabilized by weak van der Waals inter­actions.

## Related literature

For the synthesis and biological activity of 3-aza­bicyclo­[3.3.1]nonan-9-ones, see: Jeyaraman & Avila (1981 ▶); Barker *et al.* (2005 ▶); Parthiban *et al.* (2009*a* ▶, 2010*b* ▶,*c* ▶, 2011 ▶). For related structures, see: Parthiban *et al.* (2009*b* ▶,*c* ▶, 2010*a* ▶,*c* ▶); Cox *et al.* (1985 ▶); Smith-Verdier *et al.* (1983 ▶); Padegimas & Kovacic (1972 ▶). For ring puckering parameters, see: Cremer & Pople (1975 ▶); Nardelli (1983 ▶).
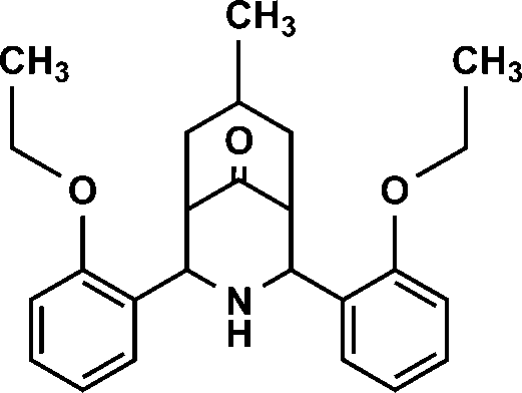


## Experimental

### 

#### Crystal data

C_25_H_31_NO_3_
*M* *_r_* = 393.51
Monoclinic, 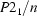

*a* = 10.3147 (6) Å
*b* = 11.8817 (6) Å
*c* = 18.7809 (10) Å
β = 100.866 (2)°
*V* = 2260.4 (2) Å^3^
*Z* = 4
Mo *K*α radiation
μ = 0.08 mm^−1^
*T* = 298 K
0.35 × 0.28 × 0.15 mm

#### Data collection

Bruker APEXII CCD area-detector diffractometer
Absorption correction: multi-scan (*SADABS*; Bruker, 2004 ▶) *T*_min_ = 0.974, *T*_max_ = 0.989
12446 measured reflections
3876 independent reflections
2415 reflections with *I* > 2σ(*I*)
*R*_int_ = 0.025

#### Refinement

*R*[*F* ^2^ > 2σ(*F* ^2^)] = 0.042
*wR*(*F* ^2^) = 0.117
*S* = 1.03
3876 reflections
269 parameters
H atoms treated by a mixture of independent and constrained refinement
Δρ_max_ = 0.12 e Å^−3^
Δρ_min_ = −0.13 e Å^−3^

### 

Data collection: *APEX2* (Bruker, 2004 ▶); cell refinement: *APEX2* and *SAINT-Plus* (Bruker, 2004 ▶); data reduction: *SAINT-Plus* and *XPREP* (Bruker, 2004 ▶); program(s) used to solve structure: *SHELXS97* (Sheldrick, 2008 ▶); program(s) used to refine structure: *SHELXL97* (Sheldrick, 2008 ▶); molecular graphics: *ORTEP-3* (Farrugia, 1997 ▶); software used to prepare material for publication: *SHELXL97*.

## Supplementary Material

Crystal structure: contains datablocks global, I. DOI: 10.1107/S1600536811018472/lw2064sup1.cif

Structure factors: contains datablocks I. DOI: 10.1107/S1600536811018472/lw2064Isup2.hkl

Supplementary material file. DOI: 10.1107/S1600536811018472/lw2064Isup3.cml

Additional supplementary materials:  crystallographic information; 3D view; checkCIF report

## Figures and Tables

**Fig. 1.**
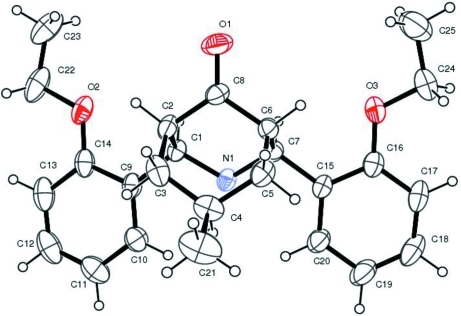
Anistropic displacement representation of the molecule with atoms represented with 30% probability ellipsoids.

## supplementary crystallographic information

### Comment

The 3-azabicycle nucleus is an important class of pharmacophore due to its broad spectrum of biological activities such as antibacterial, antimycobacterial, antifungal, anticancer, antitussive, antiinflammatory, sedative, antipyretic and calcium antagonistic activity (Jeyaraman & Avila, 1981; Barker *et al.*, 2005; Parthiban *et al.*, 2009*a*, 2010*b*, 2010*c*, 2011). Its biological significane prompted the medicinal chemists to synthesize some structural analogs. Since the stereochemistry plays an important role in biological actions, it is of immense help to establish the stereochemistry of the synthesized bio-potent molecules. Since several stereomers are possible for the synthesized title compound along with different conformations such as chair-chair (Parthiban *et al.*, 2009*b*, 2009*c*, 2010*a*; Cox *et al.*, 1985), chair-boat (Parthiban *et al.*, 2010*c*; Smith-Verdier *et al.*, 1983) and boat-boat (Padegimas & Kovacic, 1972), the title compound was undertaken for the present single-crystal XRD study to establish the stereochemistry.

The analysis of torsion angles, asymmetry parameters and puckering parameters calculated for the title compound shows that the piperidine ring slightly deviates the ideal chair conformation. According to Cremer & Pople, the total puckering amplitude, Q_T_ is 0.5889 (18) Å and the phase angle θ is 7.19 (18)° (Cremer & Pople, 1975) for the piperidine ring. Also according to Nardelli, the smallest displacement asymmetry parameters q_2_ and q_3_ are 0.0741 (18) and 0.5843 (18)°, respectively (Nardelli, 1983).

The cyclohexanone ring deviates more than the piperidone ring from the ideal chair conformation. According to Cremer and Pople the Q_T_ = 0.554 (2) and θ = 12.2 (2)° (Cremer & Pople, 1975) and by Nardelli, q_2_ = 0.118 (2) and q_3_ = 0.541 (2)° (Nardelli, 1983).

The torsion angles of C8—C6—C7—C15 and C8—C2—C1—C9 are -179.07 (14) and 176.83 (14)°, respectively.

The above detailed analysis of the title compound C_25_H_31_NO_3_, clearly shows that the compound exists in a twin-chair conformation with an equatorial orientation of the *ortho*-ethoxyphenyl units on both sides of the secondary amino group. The *ortho*-ethoxyphenyl groups are orientated at a dihedral angle of 12.41 (4)° with respect to each other. The methyl group attached to the cyclohexanone part occupies an exocyclic equatorial disposition. The crystal packing is stabilized by weak van der Waals interactions.

### Experimental

The 7-methyl-2,4-*bis*(2-ethoxyphenyl)-3-azabicyclo[3.3.1]nonan-9-one was synthesized by a modified and an optimized Mannich condensation in one-pot, using 2-ethoxybenzaldehyde (0.1 mol, 15.02 g/13.94 ml), 4-methylcyclohexanone (0.05 mol, 5.61 g/6.14 ml) and ammonium acetate (0.075 mol, 5.78 g) in a 50 ml of absolute ethanol. The mixture was gently warmed on a hot plate at 303–308 K (30–35° C) with moderate stirring till the complete consumption of the starting materials, which was monitored by TLC. At the end, the crude azabicyclic ketone was separated by filtration and gently washed with 1:5 cold ethanol-ether mixture. X-ray diffraction quality crystals of the title compound were obtained by slow evaporation from ethanol.

### Refinement

The nitrogen H atom was located in a difference Fourier map and refined isotropically. Other hydrogen atoms were fixed geometrically and allowed to ride on the parent carbon atoms with aromatic C—H = 0.93 Å, aliphatic C—H = 0.98Å and methylene C—H = 0.97 Å. The displacement parameters were set for phenyl, methylene and aliphatic H atoms at *U*_iso_(H) = 1.2*U*_eq_(C) and for methyl H atoms at *U*_iso_(H) = 1.5*U*_eq_(C)..

### Crystal data

**Table:**

C_25_H_31_NO_3_	*F*(000) = 848
*M**_r_* = 393.51	*D*_x_ = 1.156 Mg m^−^^3^
Monoclinic, *P*2_1_/*n*	Mo *K*α radiation, λ = 0.71073 Å
Hall symbol: -P 2yn	Cell parameters from 3407 reflections
*a* = 10.3147 (6) Å	θ = 2.6–21.8°
*b* = 11.8817 (6) Å	µ = 0.08 mm^−^^1^
*c* = 18.7809 (10) Å	*T* = 298 K
β = 100.866 (2)°	Block, colourless
*V* = 2260.4 (2) Å^3^	0.35 × 0.28 × 0.15 mm
*Z* = 4	

### Data collection

**Table:**

Bruker APEXII CCD area-detector diffractometer	3876 independent reflections
Radiation source: fine-focus sealed tube	2415 reflections with *I* > 2σ(*I*)
graphite	*R*_int_ = 0.025
φ and ω scans	θ_max_ = 25.6°, θ_min_ = 2.0°
Absorption correction: multi-scan (*SADABS*; Bruker, 2004)	*h* = −10→11
*T*_min_ = 0.974, *T*_max_ = 0.989	*k* = −14→14
12446 measured reflections	*l* = −22→16

### Refinement

**Table:**

Refinement on *F*^2^	Primary atom site location: structure-invariant direct methods
Least-squares matrix: full	Secondary atom site location: difference Fourier map
*R*[*F*^2^ > 2σ(*F*^2^)] = 0.042	Hydrogen site location: inferred from neighbouring sites
*wR*(*F*^2^) = 0.117	H atoms treated by a mixture of independent and constrained refinement
*S* = 1.03	*w* = 1/[σ^2^(*F*_o_^2^) + (0.047*P*)^2^ + 0.3732*P*] where *P* = (*F*_o_^2^ + 2*F*_c_^2^)/3
3876 reflections	(Δ/σ)_max_ < 0.001
269 parameters	Δρ_max_ = 0.12 e Å^−^^3^
0 restraints	Δρ_min_ = −0.13 e Å^−^^3^

### Special details

**Table:**

Geometry. All e.s.d.'s (except the e.s.d. in the dihedral angle between two l.s. planes)are estimated using the full covariance matrix. The cell e.s.d.'s are takeninto account individually in the estimation of e.s.d.'s in distances, anglesand torsion angles; correlations between e.s.d.'s in cell parameters are onlyused when they are defined by crystal symmetry. An approximate (isotropic)treatment of cell e.s.d.'s is used for estimating e.s.d.'s involving l.s. planes.
Refinement. Refinement of *F*^2^ against ALL reflections. The weighted *R*-factor *wR* and goodness of fit *S* are based on *F*^2^, conventional *R*-factors *R* are based on *F*, with *F* set to zero for negative *F*^2^. The threshold expression of *F*^2^ > σ(*F*^2^) is used only for calculating *R*-factors(gt) *etc*. and is not relevant to the choice of reflections for refinement. *R*-factors based on *F*^2^ are statistically about twice as large as those based on *F*, and *R*- factors based on ALL data will be even larger.

### Fractional atomic coordinates and isotropic or equivalent isotropic displacement parameters (Å^2^)

**Table:**

	*x*	*y*	*z*	*U*_iso_*/*U*_eq_	
C1	1.07118 (17)	0.68920 (14)	0.12439 (9)	0.0475 (4)	
H1	1.1292	0.6243	0.1379	0.057*	
C2	0.99532 (18)	0.71301 (15)	0.18699 (9)	0.0553 (5)	
H2	1.0598	0.7203	0.2323	0.066*	
C3	0.9088 (2)	0.81806 (16)	0.17738 (11)	0.0676 (6)	
H3A	0.8763	0.8312	0.2219	0.081*	
H3B	0.9629	0.8821	0.1698	0.081*	
C4	0.7921 (2)	0.81182 (16)	0.11504 (11)	0.0659 (6)	
H4	0.8258	0.8178	0.0698	0.079*	
C5	0.72060 (18)	0.70069 (16)	0.11430 (10)	0.0585 (5)	
H5A	0.6605	0.6933	0.0681	0.070*	
H5B	0.6678	0.7023	0.1519	0.070*	
C6	0.80887 (17)	0.59660 (14)	0.12573 (8)	0.0489 (4)	
H6	0.7547	0.5311	0.1324	0.059*	
C7	0.88565 (16)	0.57076 (14)	0.06386 (8)	0.0454 (4)	
H7	0.9362	0.5012	0.0759	0.054*	
C8	0.90968 (17)	0.61391 (15)	0.19325 (9)	0.0522 (5)	
C9	1.15495 (18)	0.78883 (15)	0.11170 (10)	0.0522 (5)	
C10	1.1188 (2)	0.86086 (16)	0.05364 (11)	0.0643 (5)	
H10	1.0419	0.8464	0.0202	0.077*	
C11	1.1939 (3)	0.95391 (18)	0.04389 (15)	0.0868 (7)	
H11	1.1680	1.0011	0.0042	0.104*	
C12	1.3066 (3)	0.9759 (2)	0.09300 (18)	0.0975 (8)	
H12	1.3576	1.0384	0.0866	0.117*	
C13	1.3453 (2)	0.9065 (2)	0.15186 (15)	0.0861 (7)	
H13	1.4219	0.9224	0.1852	0.103*	
C14	1.27035 (19)	0.81263 (17)	0.16149 (11)	0.0629 (5)	
C15	0.79067 (17)	0.55378 (15)	−0.00749 (9)	0.0495 (5)	
C16	0.71335 (18)	0.45680 (16)	−0.01864 (10)	0.0569 (5)	
C17	0.6211 (2)	0.4422 (2)	−0.08214 (12)	0.0741 (6)	
H17	0.5700	0.3773	−0.0892	0.089*	
C18	0.6060 (2)	0.5244 (3)	−0.13440 (12)	0.0885 (8)	
H18	0.5439	0.5150	−0.1768	0.106*	
C19	0.6809 (2)	0.6193 (2)	−0.12479 (11)	0.0871 (7)	
H19	0.6707	0.6743	−0.1607	0.105*	
C20	0.7721 (2)	0.63362 (18)	−0.06142 (10)	0.0671 (6)	
H20	0.8223	0.6991	−0.0551	0.080*	
C21	0.6966 (3)	0.9105 (2)	0.11769 (15)	0.1095 (9)	
H21A	0.6663	0.9092	0.1630	0.164*	
H21B	0.6225	0.9037	0.0785	0.164*	
H21C	0.7414	0.9803	0.1133	0.164*	
C22	1.4219 (2)	0.7531 (3)	0.26893 (13)	0.1002 (9)	
H22A	1.4220	0.8246	0.2938	0.120*	
H22B	1.4965	0.7517	0.2442	0.120*	
C23	1.4315 (3)	0.6584 (3)	0.32186 (15)	0.1209 (11)	
H23A	1.3550	0.6583	0.3441	0.181*	
H23B	1.5093	0.6676	0.3585	0.181*	
H23C	1.4365	0.5883	0.2970	0.181*	
C24	0.6380 (2)	0.29441 (18)	0.03834 (13)	0.0819 (7)	
H24A	0.6386	0.2398	0.0000	0.098*	
H24B	0.5510	0.3284	0.0315	0.098*	
C25	0.6690 (3)	0.2388 (2)	0.10965 (16)	0.1147 (10)	
H25A	0.7545	0.2041	0.1155	0.172*	
H25B	0.6037	0.1823	0.1127	0.172*	
H25C	0.6691	0.2936	0.1472	0.172*	
H1N	1.0230 (17)	0.6437 (14)	0.0263 (9)	0.054 (6)*	
N1	0.97762 (14)	0.66179 (12)	0.05778 (8)	0.0461 (4)	
O1	0.92001 (15)	0.55398 (12)	0.24612 (7)	0.0837 (5)	
O2	1.30161 (13)	0.73934 (13)	0.21804 (7)	0.0743 (4)	
O3	0.73609 (13)	0.37931 (11)	0.03597 (7)	0.0719 (4)	

### Atomic displacement parameters (Å^2^)

**Table:**

	*U*^11^	*U*^22^	*U*^33^	*U*^12^	*U*^13^	*U*^23^
C1	0.0384 (11)	0.0552 (10)	0.0466 (10)	−0.0026 (8)	0.0018 (8)	−0.0035 (8)
C2	0.0484 (12)	0.0740 (12)	0.0406 (9)	−0.0126 (10)	0.0011 (8)	−0.0080 (9)
C3	0.0705 (15)	0.0652 (13)	0.0748 (13)	−0.0188 (11)	0.0337 (12)	−0.0210 (10)
C4	0.0688 (14)	0.0615 (12)	0.0749 (13)	0.0125 (11)	0.0328 (12)	0.0059 (10)
C5	0.0434 (12)	0.0802 (13)	0.0527 (11)	0.0004 (10)	0.0108 (9)	−0.0027 (9)
C6	0.0473 (11)	0.0536 (10)	0.0453 (10)	−0.0099 (9)	0.0079 (8)	0.0020 (8)
C7	0.0407 (11)	0.0469 (9)	0.0463 (9)	−0.0020 (8)	0.0023 (8)	−0.0020 (7)
C8	0.0509 (12)	0.0644 (11)	0.0411 (10)	0.0019 (9)	0.0079 (8)	0.0055 (9)
C9	0.0403 (11)	0.0596 (11)	0.0575 (11)	−0.0062 (9)	0.0114 (9)	−0.0097 (9)
C10	0.0566 (13)	0.0667 (12)	0.0722 (13)	−0.0074 (11)	0.0190 (10)	0.0011 (10)
C11	0.0885 (19)	0.0707 (15)	0.1100 (19)	−0.0106 (14)	0.0411 (16)	0.0105 (13)
C12	0.085 (2)	0.0778 (17)	0.141 (2)	−0.0335 (15)	0.0503 (18)	−0.0152 (17)
C13	0.0608 (15)	0.0964 (18)	0.1040 (19)	−0.0290 (14)	0.0232 (13)	−0.0319 (15)
C14	0.0456 (13)	0.0740 (13)	0.0710 (13)	−0.0138 (11)	0.0159 (11)	−0.0201 (11)
C15	0.0416 (11)	0.0620 (11)	0.0441 (10)	−0.0024 (9)	0.0057 (8)	−0.0078 (9)
C16	0.0497 (12)	0.0677 (12)	0.0534 (11)	−0.0044 (10)	0.0100 (9)	−0.0124 (10)
C17	0.0576 (14)	0.0973 (16)	0.0650 (13)	−0.0179 (12)	0.0051 (11)	−0.0303 (13)
C18	0.0680 (17)	0.140 (2)	0.0504 (13)	−0.0093 (16)	−0.0059 (11)	−0.0146 (15)
C19	0.0799 (17)	0.123 (2)	0.0516 (12)	−0.0099 (16)	−0.0059 (12)	0.0122 (13)
C20	0.0629 (14)	0.0857 (14)	0.0490 (11)	−0.0103 (11)	0.0014 (10)	0.0052 (10)
C21	0.119 (2)	0.0863 (17)	0.140 (2)	0.0412 (16)	0.0676 (19)	0.0127 (16)
C22	0.0462 (15)	0.170 (3)	0.0793 (16)	−0.0167 (16)	−0.0019 (13)	−0.0290 (18)
C23	0.088 (2)	0.179 (3)	0.0807 (18)	0.021 (2)	−0.0231 (15)	−0.0037 (19)
C24	0.0777 (16)	0.0658 (13)	0.1047 (18)	−0.0212 (12)	0.0236 (14)	−0.0183 (13)
C25	0.122 (2)	0.0769 (17)	0.147 (3)	−0.0075 (16)	0.029 (2)	0.0255 (17)
N1	0.0390 (9)	0.0585 (9)	0.0413 (8)	−0.0041 (7)	0.0088 (7)	−0.0067 (7)
O1	0.0861 (11)	0.1035 (11)	0.0580 (8)	−0.0004 (9)	0.0049 (7)	0.0331 (8)
O2	0.0478 (9)	0.0991 (11)	0.0686 (9)	−0.0109 (8)	−0.0083 (7)	−0.0111 (8)
O3	0.0709 (10)	0.0583 (8)	0.0812 (9)	−0.0193 (7)	0.0010 (8)	−0.0064 (7)

### Geometric parameters (Å, °)

**Table:**

C1—N1	1.465 (2)	C13—H13	0.9300
C1—C9	1.511 (2)	C14—O2	1.364 (2)
C1—C2	1.556 (2)	C15—C20	1.375 (2)
C1—H1	0.9800	C15—C16	1.395 (2)
C2—C8	1.490 (2)	C16—O3	1.365 (2)
C2—C3	1.525 (3)	C16—C17	1.389 (3)
C2—H2	0.9800	C17—C18	1.372 (3)
C3—C4	1.515 (3)	C17—H17	0.9300
C3—H3A	0.9700	C18—C19	1.360 (3)
C3—H3B	0.9700	C18—H18	0.9300
C4—C5	1.511 (3)	C19—C20	1.381 (3)
C4—C21	1.539 (3)	C19—H19	0.9300
C4—H4	0.9800	C20—H20	0.9300
C5—C6	1.527 (2)	C21—H21A	0.9600
C5—H5A	0.9700	C21—H21B	0.9600
C5—H5B	0.9700	C21—H21C	0.9600
C6—C8	1.495 (2)	C22—O2	1.426 (2)
C6—C7	1.555 (2)	C22—C23	1.493 (4)
C6—H6	0.9800	C22—H22A	0.9700
C7—N1	1.457 (2)	C22—H22B	0.9700
C7—C15	1.517 (2)	C23—H23A	0.9600
C7—H7	0.9800	C23—H23B	0.9600
C8—O1	1.2101 (19)	C23—H23C	0.9600
C9—C10	1.381 (3)	C24—O3	1.436 (2)
C9—C14	1.397 (3)	C24—C25	1.474 (3)
C10—C11	1.382 (3)	C24—H24A	0.9700
C10—H10	0.9300	C24—H24B	0.9700
C11—C12	1.366 (3)	C25—H25A	0.9600
C11—H11	0.9300	C25—H25B	0.9600
C12—C13	1.377 (3)	C25—H25C	0.9600
C12—H12	0.9300	N1—H1N	0.848 (18)
C13—C14	1.388 (3)		
			
N1—C1—C9	110.10 (14)	C12—C13—H13	119.9
N1—C1—C2	109.96 (14)	C14—C13—H13	119.9
C9—C1—C2	111.15 (14)	O2—C14—C13	123.9 (2)
N1—C1—H1	108.5	O2—C14—C9	116.01 (17)
C9—C1—H1	108.5	C13—C14—C9	120.1 (2)
C2—C1—H1	108.5	C20—C15—C16	117.59 (16)
C8—C2—C3	108.30 (15)	C20—C15—C7	122.44 (16)
C8—C2—C1	107.82 (14)	C16—C15—C7	119.90 (15)
C3—C2—C1	115.19 (15)	O3—C16—C17	123.62 (18)
C8—C2—H2	108.5	O3—C16—C15	115.60 (15)
C3—C2—H2	108.5	C17—C16—C15	120.78 (19)
C1—C2—H2	108.5	C18—C17—C16	119.5 (2)
C4—C3—C2	114.42 (15)	C18—C17—H17	120.2
C4—C3—H3A	108.7	C16—C17—H17	120.2
C2—C3—H3A	108.7	C19—C18—C17	120.7 (2)
C4—C3—H3B	108.7	C19—C18—H18	119.7
C2—C3—H3B	108.7	C17—C18—H18	119.7
H3A—C3—H3B	107.6	C18—C19—C20	119.6 (2)
C5—C4—C3	111.43 (15)	C18—C19—H19	120.2
C5—C4—C21	110.60 (18)	C20—C19—H19	120.2
C3—C4—C21	110.98 (18)	C15—C20—C19	121.9 (2)
C5—C4—H4	107.9	C15—C20—H20	119.1
C3—C4—H4	107.9	C19—C20—H20	119.1
C21—C4—H4	107.9	C4—C21—H21A	109.5
C4—C5—C6	115.42 (15)	C4—C21—H21B	109.5
C4—C5—H5A	108.4	H21A—C21—H21B	109.5
C6—C5—H5A	108.4	C4—C21—H21C	109.5
C4—C5—H5B	108.4	H21A—C21—H21C	109.5
C6—C5—H5B	108.4	H21B—C21—H21C	109.5
H5A—C5—H5B	107.5	O2—C22—C23	107.5 (2)
C8—C6—C5	107.99 (14)	O2—C22—H22A	110.2
C8—C6—C7	106.87 (14)	C23—C22—H22A	110.2
C5—C6—C7	115.41 (14)	O2—C22—H22B	110.2
C8—C6—H6	108.8	C23—C22—H22B	110.2
C5—C6—H6	108.8	H22A—C22—H22B	108.5
C7—C6—H6	108.8	C22—C23—H23A	109.5
N1—C7—C15	110.55 (13)	C22—C23—H23B	109.5
N1—C7—C6	110.04 (13)	H23A—C23—H23B	109.5
C15—C7—C6	110.56 (13)	C22—C23—H23C	109.5
N1—C7—H7	108.5	H23A—C23—H23C	109.5
C15—C7—H7	108.5	H23B—C23—H23C	109.5
C6—C7—H7	108.5	O3—C24—C25	108.05 (19)
O1—C8—C2	124.61 (16)	O3—C24—H24A	110.1
O1—C8—C6	123.69 (17)	C25—C24—H24A	110.1
C2—C8—C6	111.70 (14)	O3—C24—H24B	110.1
C10—C9—C14	118.18 (18)	C25—C24—H24B	110.1
C10—C9—C1	122.21 (16)	H24A—C24—H24B	108.4
C14—C9—C1	119.57 (17)	C24—C25—H25A	109.5
C9—C10—C11	121.7 (2)	C24—C25—H25B	109.5
C9—C10—H10	119.1	H25A—C25—H25B	109.5
C11—C10—H10	119.1	C24—C25—H25C	109.5
C12—C11—C10	119.4 (2)	H25A—C25—H25C	109.5
C12—C11—H11	120.3	H25B—C25—H25C	109.5
C10—C11—H11	120.3	C7—N1—C1	115.58 (13)
C11—C12—C13	120.6 (2)	C7—N1—H1N	108.7 (12)
C11—C12—H12	119.7	C1—N1—H1N	106.8 (12)
C13—C12—H12	119.7	C14—O2—C22	119.78 (18)
C12—C13—C14	120.1 (2)	C16—O3—C24	118.36 (16)
			
N1—C1—C2—C8	54.66 (18)	C11—C12—C13—C14	0.5 (4)
C9—C1—C2—C8	176.83 (14)	C12—C13—C14—O2	179.8 (2)
N1—C1—C2—C3	−66.38 (19)	C12—C13—C14—C9	−0.4 (3)
C9—C1—C2—C3	55.80 (19)	C10—C9—C14—O2	179.74 (17)
C8—C2—C3—C4	−54.3 (2)	C1—C9—C14—O2	2.3 (2)
C1—C2—C3—C4	66.4 (2)	C10—C9—C14—C13	−0.1 (3)
C2—C3—C4—C5	45.8 (2)	C1—C9—C14—C13	−177.49 (17)
C2—C3—C4—C21	169.49 (17)	N1—C7—C15—C20	−17.0 (2)
C3—C4—C5—C6	−45.3 (2)	C6—C7—C15—C20	105.11 (19)
C21—C4—C5—C6	−169.20 (17)	N1—C7—C15—C16	166.35 (16)
C4—C5—C6—C8	52.7 (2)	C6—C7—C15—C16	−71.5 (2)
C4—C5—C6—C7	−66.8 (2)	C20—C15—C16—O3	179.34 (17)
C8—C6—C7—N1	−56.66 (17)	C7—C15—C16—O3	−3.9 (2)
C5—C6—C7—N1	63.43 (17)	C20—C15—C16—C17	0.0 (3)
C8—C6—C7—C15	−179.07 (14)	C7—C15—C16—C17	176.80 (16)
C5—C6—C7—C15	−58.98 (18)	O3—C16—C17—C18	−179.4 (2)
C3—C2—C8—O1	−117.4 (2)	C15—C16—C17—C18	−0.1 (3)
C1—C2—C8—O1	117.36 (19)	C16—C17—C18—C19	0.4 (4)
C3—C2—C8—C6	62.90 (18)	C17—C18—C19—C20	−0.7 (4)
C1—C2—C8—C6	−62.34 (18)	C16—C15—C20—C19	−0.2 (3)
C5—C6—C8—O1	118.6 (2)	C7—C15—C20—C19	−176.98 (18)
C7—C6—C8—O1	−116.67 (19)	C18—C19—C20—C15	0.6 (3)
C5—C6—C8—C2	−61.71 (19)	C15—C7—N1—C1	177.62 (14)
C7—C6—C8—C2	63.03 (18)	C6—C7—N1—C1	55.21 (18)
N1—C1—C9—C10	18.0 (2)	C9—C1—N1—C7	−176.73 (14)
C2—C1—C9—C10	−104.10 (19)	C2—C1—N1—C7	−53.93 (19)
N1—C1—C9—C14	−164.69 (16)	C13—C14—O2—C22	−4.1 (3)
C2—C1—C9—C14	73.2 (2)	C9—C14—O2—C22	176.08 (18)
C14—C9—C10—C11	0.4 (3)	C23—C22—O2—C14	−177.42 (19)
C1—C9—C10—C11	177.78 (18)	C17—C16—O3—C24	−18.1 (3)
C9—C10—C11—C12	−0.3 (3)	C15—C16—O3—C24	162.60 (17)
C10—C11—C12—C13	−0.1 (4)	C25—C24—O3—C16	−167.01 (19)
